# Acromial Tilt, Lateral Acromial Angle, and Acromiohumeral Interval as Risk Factors for Full-Thickness Supraspinatus Tendon Ruptures

**DOI:** 10.7759/cureus.73370

**Published:** 2024-11-10

**Authors:** Thomas Caffard, Marius Ludwig, Thomas Kappe, Heiko Reichel, Mirco Sgroi

**Affiliations:** 1 Department of Orthopedic Surgery, University of Ulm, Ulm, DEU

**Keywords:** acromiohumeral distance, critical shoulder angle, lateral acromial angle, rotator cuff tear, supraspinatus tendon

## Abstract

Introduction

The aim of this study was to investigate whether the morphology of the acromion and the inclination of the glenoid are associated with the risk of supraspinatus (SSP) tendon ruptures.

Materials and methods

A total of 106 patients were enrolled in this study between August 2012 and February 2014, including 55 symptomatic patients with an SSP tendon rupture (ruptured group) and 51 patients with an intact SSP (control group). MRI of the shoulder was performed for all patients in both groups. All MR images were analyzed by two blinded observers to measure the acromiohumeral interval (AHI), critical shoulder angle (CSA), acromial slope (AS), acromial tilt (AT), lateral acromial angle (LAA), acromion index (AI), and glenoid inclination (GI). Furthermore, both observers analyzed tendon integrity and quality on all MRIs in both groups. The results of the radiological examination concerning acromial and glenoidal morphology were compared between the control group with intact SSP tendons and the rupture group.

Results

Patients with an SSP tendon rupture had a narrower AHI (9.1 ± 1.4 mm vs. 7.8 ± 2.1 mm; p < 0.01), a greater AT (36.0 ± 5.4° vs. 39.7 ± 5.9°; p < 0.01), and a lower LAA (81.1 ± 7.2° vs. 76.2 ± 5.0°; p < 0.01). Patients with an AHI smaller than 8.2 mm (OR 1.88 [95% CI 1.2 to 2.7]; p < 0.01) or an AT greater than 36.5° (OR 3.56 [95% CI 1.57 to 8.01]; p = 0.03) or a LAA lower than 80.5° (OR 4.04 [95% CI 2.04 to 7.90]; p < 0.01) had higher risk for an SSP tendon rupture. No differences between either group were found in relation to the AS, CSA, AI, or glenoid inclination.

Conclusions

The results of this study showed that the AHI, LAA and AT correlated with SSP tendon rupture. It should be noted that the preoperative AHI less than 8.2 mm, AT greater than 36.5° or LAA less than 80.5° may be associated with SSP tendon ruptures.

## Introduction

Rotator cuff ruptures are one of the most common causes of pain and limited range of motion of the shoulder [1]. Ruptures of the supraspinatus (SSP) tendon are very common. Their prevalence is estimated to be between 5% and 40% [2]. The consequences of SSP tendon ruptures have been extensively investigated and are well known [3]. However, despite their central epidemiological importance, the pathogenesis of rotator cuff ruptures remains controversial [4,5]. The morphology of the acromion and the glenoidal inclination (GI) were postulated as possible causes of SSP tendon ruptures [6]. A greater ratio of glenohumeral joint shear to joint compression forces and larger deltoid activity due to a more pronounced lateral extension of the acromion and the resulting overload of the SSP tendon has been proposed as an explanation for this association [7]. Several studies have attempted to concretize this theoretical relationship statistically, and various radiological parameters have therefore been developed to measure the lateral extent of the acromion and the GI, such as the critical shoulder angle (CSA) [5,8]. As a result, many studies have investigated a possible association between these radiological measurements and the risk of complete rotator cuff rupture [9,10]. Several studies showed that the CSA was a risk factor for an SSP tendon rupture [6,8]. However, other research studies were not able to confirm this association [11]. Among several studies, conflicting results have been obtained and no morphological parameter has been established as a definitive risk factor in clinical practice [12]. Thus, it remains unclear whether a more pronounced lateral extension of the acromion is associated with a greater risk of suffering SSP tendon ruptures. To improve our understanding of the pathogenesis of SSP tendon ruptures, a comparison between patients with intact and ruptured SSP tendons in relation to lateral extension of the acromion may be helpful. Moreover, it would be interesting to know whether greater inclination of the glenoid could influence the risk of suffering an SSP tendon rupture. We therefore asked: (1) Is there an association between a greater lateral extension and descending inclination of the acromion and the risk of suffering an SSP tendon rupture? (2) Does greater GI lead to a higher risk of SSP tendon rupture? The null hypothesis was that the morphology of the acromion and the inclination of the glenoid are not associated with the risk of SSP tendon rupture.

## Materials and methods

Study design and setting

This was a retrospective study investigating the association between acromial morphology, GI and the risk of suffering an SSP tendon rupture. The acromial morphology and glenoidal inclination of patients with a symptomatic rupture of the SSP tendon were compared with patients with an intact SSP tendon. This study was performed at the Department of Orthopedic Surgery, University of Ulm in Germany and involved two observers (TC and ML), who were residents in orthopedics.

Participants

Between August 2012 and February 2014, 63 patients with chronic full-thickness SSP tendon ruptures were considered for inclusion in the present study. The inclusion criterion was the presence of a full-thickness rupture of the SSP tendon. The exclusion criteria were partial rupture of the SSP tendon, poor quality of preoperative MRI, and age under 18 years. Based on these criteria, 8% (five of 63) were excluded because they had a partial rupture, 5% (three of 63) were excluded because of missing MRI planes or slices (such as sagittal, axial, or frontal), leaving 87% (55 of 63) for inclusion in the present analysis. Furthermore, we included 51 patients with an intact SSP tendon as the control group. Therefore, a total of 106 patients were enrolled in this study, including 55 patients with an SSP tendon rupture and 51 patients with an intact SSP. 

Descriptive data

In the rupture group, a total of 45% (25 of 55) of the patients were women. The mean age was 66.5 ± 9.9 years (range: 46 - 80 years). In the control group, a total of 51% (26 of 51) of the patients were women. The mean age was 60.0 ± 10.1 years (range: 36 - 80 years). The right side was affected in 67% (37 of 55) of the patients in the rupture group with a mean BMI of 29 ± 5 kg/m^2^ (range: 21 - 44 kg/m^2^). The right side was affected in 25% (13 of 51) of patients in the control group with a mean BMI of 28 ± 5 kg/m^2^ (range: 17 - 39 kg/m^2^). 

Radiological examination 

MRI of the shoulder was performed on all patients in both groups. MRI was performed using a 1.5 Tesla MRI scanner (Magnetom TIM-Symphony). The patients were positioned in the supine position with the arm in neutral rotation by the side of their body. A dedicated standard shoulder coil was placed over the shoulder. Two blinded clinicians (TC, ML) analyzed MR images independently from one another. Both observers received written and verbal training on the measurements to be carried out prior to the measurement. They performed several trial measurements and only started the real measurement when excellent intrarater reliability (intraclass correlation coefficient > 0.9) was achieved. To obtain intrarater and interrater correlations, two orthopedic residents (TC, ML) repeated all measurements; one of them (TC), who was blinded to the first measurements, took repeated measurements after at least 6 weeks. To assess acromial morphology, we measured the following parameters in both groups: acromiohumeral interval (AHI), CSA, acromial slope (AS), acromial tilt (AT), lateral acromial angle (LAA), acromion index (AI), and GI.

Acromiohumeral Interval 

The AHI was defined as the shortest distance between the inferior cortex of the acromion and the top of the humeral head. In the MRI, two parallel auxiliary lines were drawn for the measurement: the upper auxiliary line was placed on the lower edge of the acromion and the lower auxiliary line was placed tangentially to the osseous humeral head [13]. We measured the acromiohumeral interval because it is widely used in clinical practice to indicate the superior migration of the humeral head.

Critical Shoulder Angle 

The CSA was measured at the coronal-oblique view. A line was drawn between the upper and lower edges of the glenoid. The most lateral point of the acromial border was then identified and a line was drawn connecting the inferior border of the glenoid to the lateral border of the acromion [8]. The CSA is frequently utilized as a parameter to measure the lateral extent of the acromion and the resulting GI.

Acromial Slope

The AS was determined by the angle formed by a line from the foremost point of the acromion to the middle point of the undersurface of the acromion and a line between the most posterior point of the undersurface of the acromion and the middle point of the undersurface of the acromion [14]. 

Acromial Tilt 

The AT was defined as the angle formed by a line connecting the most posterior point of the inferior surface of the acromion and the most anterior point of the acromion and a line extending from the most posterior point of the acromion to the tip of the coracoid process. Like the AS, AT is an index of acromial inferior protrusion.

Acromial Index

To assess the AI, a line was drawn between the superior and inferior borders of the glenoid and was defined as the glenoid plane. The acromial plane was defined by drawing a line parallel to the glenoid plane at the most lateral point of the acromion. Finally, the humeral head plane was posed, parallel to the glenoid plane, at the most lateral point of the humeral head. The acromial index was calculated by dividing the distance between the glenoid and acromion planes from that between the glenoid and humeral plane. AI is a well-established parameter and represents the lateral prominence of acromion.

Lateral Acromial Angle

The LAA was defined as the angle formed by a line between the superior and inferior rims of the glenoid and a line parallel and tangential to the inferior surface of the acromion. LAA is a widely used parameter and represents the inclination of acromion.

Glenoidal Inclination

GI was measured in the coronal plane using MRI according to Hughes et al. [15]. GI was measured by defining the angle between a line parallel and tangential to the SSP fossa and a line connecting the superior and inferior margins of the glenoid. We measured the GI because it is widely used in clinical practice and is important for the biomechanics of the glenohumeral joint.

Primary and secondary study outcomes

The primary study goal was to investigate the association of acromion morphology and SSP tendon rupture. All preoperative MR images were used by two blinded observers (TC, ML) to measure the AHI, CS, AS, AT, LAA, and AI in all patients. Furthermore, both observers analyzed tendon integrity and quality on preoperative MRIs based on the Sugaya classification [16] and Castricini et al. [17], muscle atrophy according to Thomazeau et al. [18], and fatty degeneration according to Goutallier et al. [19]. The results of the radiological examination were compared with a control group consisting of 51 patients with an intact SSP tendon confirmed by MRI.

The secondary study outcome was to analyze the association between GI and the risk of suffering a full-thickness SSP-tendon rupture. Thus, on all preoperative MR images, GI was determined in all patients by two blinded observers (TC, ML). The results of the radiological examination were compared between patients with full-thickness SSP-tendon ruptures and the control group. 

Ethical approval

Institutional review board approval was obtained from the ethics committee of the University of Ulm (number 104/17). 

Statistical analysis

The statistical analysis was performed using IBM SPSS Statistics for Windows, Version 24 (Released 2016; IBM Corp., Armonk, New York, United States). The sample size was calculated based on the LAA, assuming a 95% confidence interval, and a 0.83 effect size, resulting in a sample size of at least 106 patients, with a power of 0.9. All collected data were secured in a computerized database. Descriptive statistics were used to better analyze baseline data. As already mentioned, patients were split into two groups: 1) patients with SSP tendon ruptures; 2) patients with intact SSP tendons (control group). Acromial morphology and GI measured on the preoperative MRI were compared between the two groups. Receiver operating characteristic curves were used to calculate the cutoff points for the radiologic parameters that showed significant differences between both groups. The cut-offs were calculated on the ROC curve and the point on the curve closest to (0.1) was defined as a criterion for optimality. With the obtained cut-offs sensitivity, specificity, odds ratio, and area under the curve were calculated for the AHI, AT, and LAA, to avoid bias, bootstrapping for performed. When comparing measurement methods between the intact and ruptured SSP groups, a two-sample t-test was used for interval-scaled parameters. The Wilcoxon signed-rank test was used as a t-test for ordinal-scaled dimensions. Differences were considered significant for p-values < 0.05. The intrarater reliability was evaluated with a Pearson correlation coefficient for interval-scaled measurements. The interrater reliability was calculated using the intraclass correlation coefficient (ICC). ICCs were interpreted as follows: less than 0.5 is poor, between 0.5 and 0.75 is moderate, between 0.75 and 0.90 is good, and more than 0.90 is excellent. 

## Results

Acromial morphology and SSP tendon rupture risk

Patients with an SSP tendon rupture had a narrower AHI (9.1 ± 1.4 mm vs. 7.8 ± 2.1 mm; mean difference 1.3 mm [95% CI 6.2 - 20.4]; p < 0.01), a greater AT (36.0 ± 5.4° vs. 39.7 ± 5.9°; mean difference 3.67° [95% CI 1.5-5.9]; p < 0.01), and a lower LAA (81.1 ± 7.2° vs. 76.2 ± 5.0°; mean difference 4.95° [95% CI 2.9 - 7.0]; p < 0.01) (Table 1).

**Table 1:** Comparison between patients with SSP tear and intact SSP regarding acromial morphology. AS: acromial slope (degrees ± SD); AT: acromial tilt (degrees ± SD); LAA: lateral acromial angle (degrees ± SD); AI: acromial index (ratio ± SD); AHI: acromiohumeral interval (millimeters ± SD); CSA: critical shoulder angle (degrees ± SD); CI: confidence interval; SD: standard deviation; mm: millimeters; SSP: supraspinatus. A p-value of p<0.05 was considered significant. The statistical test used was the t-test.

Variable	Intact	Ruptured	Difference	95% CI	p-value	t-value
AS, ° ± SD	25.1 ± 5.2	25.0 ± 5.3	0.64	-1.9 - 2.1	0.95	0.63
AT, ° ± SD	36.0 ± 5.4	39.7 ± 5.9	3.67	- 5.9 - 1.5	< 0.01	3.31
LAA, ° ± SD	81.1 ± 7.2	76.2 ± 5.0	4.95	2.9 - 7.0	< 0.01	2.11
AI, ratio ± SD	0.66 ± 0.07	0.67 ± 0.1	0.01	-0.04 - 0.02	0.63	0.47
AHD, mm ± SD	91.5 ± 14.9	78.2 ± 21.6	13.3	6.2 - 20.4	< 0.01	3.67
CSA, ° ± SD	34.1 ± 4.7	33.8 ± 4.4	0.33	-1.4 - 2.0	0.71	0.38
GI, ° ± SD	6.4 ± 4.0	6.2 ± 3.6	0.21	-1.2 - 1.7	0.77	0.29

A cut-off of 36.5° for the AT, 8.2 mm for the AHI, and 80.5° for the LAA were found. Patients with an AHI smaller than 8.2 mm or an AT greater than 36.5° or an LAA lower than 80.5° had higher odds for a rupture of the SSP. Sensitivity, specificity, and area under the curve of AT, AHI, and LAA are reported in Table 2. Bootstrapping was additionally performed and confirmed the results in Table 2. No differences between both groups were found in relation to AS, AI, and CSA.

**Table 2:** Sensitivity, specificity, odds ratio, and area under the curve of AT, LAA, and AHI. AHI: acromiohumeral interval; AT: acromial tilt; LAA: lateral acromial angle; CI: confidence interval. Bootstrapping confirmed the results above for AHI, AT and LAA. Sensitivity and specificity were expressed as percentages (95% CI). The odds ratio and AUC were expressed in N (95% CI). A p-value of p<0.05 was considered significant. The statistical test used was the Pearson´s chi-squared test.

Variable	AHI		AT	LAA
Sensitivity, %, (95% CI)	61 (47 - 74)	75 (65 - 88)		85 (73 - 93)
Specificity, % (95% CI)	70 (56 - 82)	55 (35 - 63)		59 (44 - 72)
Odds ratio, (95% CI)	1.88 (1.2 - 2.7)	3.56 (1.57–8.01)		4.04 (2.04–7.90)
AUC (95% CI)	0.68 (0.58–0.79)	0.67 (0.56–0.77)		0.76 (0.67 - 0.85)
p-Value	< 0.01	0.03		< 0.01
Chi-squared value	11.18	8.61		22.56

Glenoid morphology and SSP tendon rupture risk

There was no difference in glenoid inclination between patients with torn SSP tendons and the control group (6.4º ± 4.0° vs. 6.2º ± 3.6°, mean difference 0.21° [95% CI -1.2º - 1.7º]; p = 0.77).

Inter- and intrarater reliabilities

Pearson correlation coefficients and ICC revealed a good to excellent intra- and interobserver agreement (Table 3).

**Table 3:** Intra- and interrater reliabilities. AS: acromial slope; AT: acromial tilt; LAA: lateral acromial angle; AI: acromial index; AHI: acromiohumeral interval; CSA: critical shoulder angle; GI: glenoid inclination; CI: confidence interval. The intra-rater reliability was calculated using the Pearson correlation coefficient for interval-scaled measurements and was presented as N ± 95% CI. The inter-rater reliability was calculated using the intraclass correlation coefficient (ICC) and was presented as N ± 95%.

Variable	Intrarater	95% CI	p-value	Interrater	95% CI	p-value
AS	0.81	0.72 - 0.86	< 0.01	0.92	0.88 - 0.95	< 0.01
AT	0.84	0.73 - 0.90	< 0.01	0.89	0.83 - 0.93	< 0.01
LAA	0.88	0.83 - 0.92	< 0.01	0.95	0.91 - 0.96	< 0.01
AI	0.94	0.91 - 0.96	< 0.01	0.94	0.91 - 0.97	< 0.01
AHI	0.97	0.96 - 0.98	< 0.01	0.98	0.97 - 0.99	< 0.01
CSA	0.92	0.88 - 0.94	< 0.01	0.96	0.94 - 0.98	< 0.01
GI	0.91	0.88 - 0.94	< 0.01	0.93	0.89 - 0.96	< 0.01

## Discussion

An association between acromial morphology as well as glenoidal inclination and an increased rupture rate of the SSP has been postulated. However, which parameters represent a cause for SSP tendon ruptures compared to a healthy collective has not been conclusively clarified. This study showed that an AHI of less than 8.2 mm, an AT of more than 36.5° or an LAA of less than 80.5° are correlated to SSP tendon ruptures. In contrast, there was no association between glenoid inclination and SSP tendon rupture in the present study. 

Acromial morphology and SSP-rupture risk

We found a higher AT, lower AHI, and lower LAA in patients with full-thickness SSP tendon ruptures compared to patients with intact SSP tendon. This finding is important because it demonstrates that acromial morphology is correlated with rotator cuff ruptures. It should be kept in mind that acromial morphology and AHI must be considered during indication and preoperative preparation as well as in the planning of the surgical procedure, especially in the context of planned acromioplasty. Balke et al. measured an AT in asymptomatic, impingement and RCT patients of 29°, 33° and 34° respectively and demonstrated a relevant difference between the asymptomatic group and the other two groups [20]. Li et al. determined the AT on a three-dimensional CT scan and reported an AT of 29° in patients with subacromial impingement and of 23° on the asymptomatic contralateral side [21]. Prato et al. [22] found that greater AT is associated with a higher likelihood of RCTs. Balke et al. analyzed the role of AT in patients with calcifying tendinitis and compared them with patients with subacromial syndrome and found greater AT in the calcifying group [23]. In a cadaveric study, Zuckerman et al. found AT values in shoulders with RCTs and in patients with an intact rotator cuff of 28.5° and of 33.5°, respectively [24]. Concerning the AHI, the vast majority of studies performed reported that AHI plays a key role in the pathogenesis of rotator cuff ruptures [25,26]. Saupe et al. demonstrated that full-thickness SSP tendon ruptures were present in 90% of the patients with an AHI ≤ 7 mm [25]. Kaur et al. showed a mean AHI of 7.1 mm in patients with complete RCT and a mean AHI of 7.8 mm in patients with partial RCT [26]. Werner et al. believed that the cut-off point for AHI on MRI should be ≤ 6 mm [27]. Liu et al. found no association between AHI and degenerative articular-sided partial ruptures of the RC [10]. Balke et al. reported that patients with degenerative RCT presented a narrower acromial space compared to traumatic rotator cuff ruptures [6]. Regarding the LAA, two studies [26,28], conducted by Kaur et al. and Banas et al., observed that an extremely low LAA of less than 70° was only present in cases of cuff ruptures. Banas et al. specifically reported that among their participants, eight patients with complete ruptures had LAA measurements below 70° and an average age of 54 years, whereas the remaining 18 patients with rotator cuff ruptures and LAA measurements greater than 70° had an average age of 70 years [28]. Kaur et al. demonstrated a moderate correlation between LAA and age, similar to the original study by Banas et al., with a correlation coefficient of 0.46 [26,28]. The results of the present study concerning the AT and the AHI agree with the research mentioned above. However, the respectively reported cut offs are not identical between the mentioned studies. The AT and LAA measured in the present study were higher than those in the studies discussed above. This may be due to the different contexts in which the present study was conducted and especially the limited number of patients analyzed. Future studies should be performed to confirm our cut-offs values and to investigate whether acromioplasty is able to alter AT and AHI. It should then be investigated whether alteration of AT and AHI is also associated with better postoperative clinical and radiological outcomes. 

Glenoid morphology and SSP tendon rupture risk

We found that GI is not correlated with an SSP tendon rupture. This finding is important because GI was postulated to be able to modify the forces acting on the SSP tendon causing an overload. However, according to our results, no difference between patients with intact and ruptured SSP was observed. Therefore, preoperative measurement of GI is not recommended. A study compared shoulders with torn SSP tendons with the intact contralateral side and reported that the inclination angle was greater in shoulders having full-thickness rotator cuff ruptures than in shoulders without ruptures [15]. Kandemir et al. found no differences in glenoid inclination or glenoid version from shoulders with an intact rotator cuff and those with a full-thickness rupture [29]. Another study reported an association between cranial orientation of the glenoid, scapular spine, and/or decreased subacromial space with rotator cuff ruptures [5,29]. The results of the present study reflect the findings of Kandemir et al. Since the study by Kandemir et al. was a cadaver study and our study was conducted in a clinical setting, the present study represents one of the few available clinical extensions and confirmations of the study by Kandemir et al. On the other hand, a comparison between our study and the above study regarding the glenoid version is limited. This is because the aim of our study has rarely been investigated. Future studies with a greater number of involved patients are needed to confirm or contradict our results. 

Limitations

First, a limited number of patients was analyzed. To avoid bias, the included patients were subjected to a strict preselection process. The few studies conducted to date have generally not examined a significantly larger number of patients. Furthermore, the sample size calculation in this study showed sufficient power and indicated the study had sufficient validity. Therefore, this limitation should not have influenced the conclusions of our study. Second, because this was a retrospective study, standardization of imaging techniques was not possible. This is a limitation of the present study because if the MRIs of both groups did not have the same slice orientation, this could have biased the accuracy of the measurements. However, considering that most patients in clinical practice usually present with an MRI examination that has already been performed externally with varying degrees of standardization, this limitation is put into perspective. Thirdly, MRI was performed with a 1.5 Tesla device rather than a 3.0 Tesla MRI device. A 3.0 Tesla MRI would have further increased the resolution of the MRIs and possibly improved the precision of the measurements. Nevertheless, a particularly good resolution is not required to conduct the measurements of the glenoid inclination and acromial morphology; moreover, we do not have a 3.0-tesla MRI in our department, which is the same situation in most hospitals and therefore, in our opinion, not a major limitation. Fourthly, the measurements were not performed by radiologists, but by orthopedic residents. This could have led to inaccurate results. To avoid this, both observers performed several trial measurements with appropriate repetitions following verbal and written instructions. Observers began real measurements after their respective measurements were reliable. Therefore, this limitation is unlikely to influence data validity. Fifthly, the present study was carried out in a clinic in Germany and is, therefore, monocentric in design. Given the morphological nature of the study, the lack of a multicenter setting leads to a certain geographical limitation. A multicenter setting with clinics from other geographical regions of the world would have improved the representativeness of the study. On the other hand, it should be noted that few studies were able to fully reflect the geographical variance of the population, but this limitation must be taken into account when interpreting the study results. Finally, in the present study, one of the two observers (as opposed to both) repeated the measurements. This may have influenced the reliability of the results. Nonetheless, both observers were trained to perform the measurements and already have demonstrated good reliability before the real measurements. Therefore, repeating the measurements by the second observer might not have yielded any concrete benefits. Thus, this limitation is not to be considered relevant. 

## Conclusions

Given the geographical limitations of the present study, we found that AHI, LAA and AT were associated with the risk of SSP tendon rupture. In addition, we did not observe an association between GI and SSP tendon rupture. The results of our study suggest that AHI, LAA, and AT may play an important role in the pathogenesis of SSP tendon ruptures. Studies with larger numbers of patients and a wider geographical area should be conducted to clarify whether the associations observed in our study persist. In addition, studies could investigate whether surgical modification of acromial morphology during rotator cuff repair is beneficial.
